# A Review of Cognitive Changes During Acute Aerobic Exercise

**DOI:** 10.3389/fpsyg.2021.653158

**Published:** 2021-12-16

**Authors:** Julie A. Cantelon, Grace E. Giles

**Affiliations:** ^1^U.S. Army Combat Capabilities Development Command Soldier Center, Natick, MA, United States; ^2^Center for Applied Brain and Cognitive Sciences, Tufts University, Medford, MA, United States; ^3^Department of Psychology, Tufts University, Medford, MA, United States

**Keywords:** exercise, cognition, executive function, physical activity, review

## Abstract

A growing body of work has investigated the effects of acute, or single bouts of, aerobic exercise on cognitive function. However, review of this research has largely focused on changes following exercise, with less focus on cognitive changes during exercise. The purpose of this review is to discuss the critical characteristics of this literature to date, including: (1) what has been done, (2) what has been found, and (3) what is next. Furthermore, previous meta-analytic reviews have demonstrated there is a small positive effect on cognition when measured during exercise, with executive functions showing the largest effects. However, these reviews group executive functions together. Here we explore how inhibition, working memory and cognitive flexibility are individually impacted by factors such as exercise intensity or duration. Searches of electronic databases and reference lists from relevant studies resulted in 73 studies meeting inclusion criteria. Studies were grouped by executive and non-executive cognitive domains, intensity and duration of exercise bouts. Within the executive domain, we found that effects on working memory and cognitive flexibility remain mixed, effects on inhibition are clearer. Moderate intensity exercise improves response time, vigorous intensity impairs accuracy. Moderate to vigorous intensity improves response time across non-executive domains of attention, motor speed and information processing, with no significant effects on accuracy. Memory processes are consistently improved during exercise. Effects of exercise duration on response time and accuracy are nuanced and vary by cognitive domain. Studies typically explore durations of 45 min or less, extended exercise durations remain largely unexplored. We highlight factors to consider when assessing exercise-cognition relationships, as well as current gaps and future directions for work in this field.

## Introduction

Over the past decade research examining the relationship between exercise and cognition has grown tremendously. It is now widely accepted that long-term, or chronic, exercise can benefit both mental and physical health, including brain and cognitive function (Piercy et al., 2018; Erickson et al., 2019). More recently, research has begun to examine the effects of acute, or single bouts, of exercise on cognitive function. The majority of previous work examining such acute effects have compared cognitive changes before and after bouts of physical activity, with less focus on changes evoked during physical activity. However, understanding how physical activity, or “exercise,” as it is often referred to colloquially, impacts cognitive function is critical in a number of applied settings. For instance, individuals with high-stress jobs (e.g., law enforcement and military personnel) often operate under physically demanding conditions while making critical, life or death decisions. Furthermore, athletes are tasked with decision-making, pacing strategies, and emotion regulation during competition (Hyland-Monks et al., 2018).

Research has begun to demonstrate how cognition is impacted during exercise. Indeed, previous meta-analytic and integrative reviews have identified several moderating variables influencing cognitive changes during exercise. Specifically, cognitive domain, exercise intensity, duration, modality and an individual’s fitness level have all been shown to influence cognitive performance (McMorris and Graydon, 2000; Brisswalter et al., 2002; Tomporowski, 2003; Lambourne and Tomporowski, 2010; Dietrich and Audiffren, 2011; Chang et al., 2012; Davranche et al., 2015). Furthermore, timing of cognitive task administration also impacts the direction of effects. For example, task administration in the initial 20 min of exercise has been shown to result in detrimental or negligible effects, whereas task administration after 20 min of exercise results in beneficial effects. Finally, tasks categorized as measures of executive function show beneficial and significantly larger effects than any other domain of cognitive tasks (e.g., information processing, simple and choice reaction time, attention, memory) (Lambourne and Tomporowski, 2010; Chang et al., 2012).

Despite heterogeneity in empirical findings, there are clear theoretical predictions to explain changes in cognitive function during acute exercise. Early conceptualizations of acute exercise-cognition interactions were built upon arousal-performance theories (Yerkes and Dodson, 1908; Easterbrook, 1959). These posit that exercise-induced increases in physiological arousal and catecholamine activity (e.g., dopamine and norepinephrine) alter cognitive performance in an inverted-*U* pattern (McMorris et al., 2011; McMorris and Hale, 2012). For instance, improved memory in particular has been associated with increased dopamine and norepinephrine, as well as brain derived neurotrophic factor (BDNF) (Roig et al., 2013). Here, moderate-intensity exercise would enhance cognitive performance more than low- or high-intensity exercise. This early work largely focused on lower level sensory and perceptual processes, such as motor speed and information processing (McMorris and Graydon, 2000; Tomporowski, 2003). However, it has been argued that higher-order cognitive processes are more likely to be affected by exercise, particularly when assessed during exercise (McMorris and Graydon, 2000; Dietrich, 2003). These processes involve executive functions, also termed cognitive control, and include inhibition, working memory, and cognitive flexibility (Miyake et al., 2000). The Transient Hypofrontality Theory (THT) was developed to explain exercise effects on these higher-order processes. The THT holds that exercise recruits activity in motor pathways (e.g., primary and secondary motor cortices, basal ganglia, cerebellum) as well as sensory (e.g., primary sensory cortex) and autonomic pathways (e.g., hypothalamus) at the expense of structures supporting higher-order cognitive processing, including the prefrontal cortex (PFC) (Dietrich, 2006). The more recent Reticular-Activating Hypofrontality (RAH) model expands on this concept, stating that exercise activates the arousal-related reticular activating system, which benefits cognition up to certain exercise intensities, at which point exercise deactivates the prefrontal cortex leading to impairments in executive function (Dietrich and Audiffren, 2011). Taken together, current theories suggest that increasing exercise intensity or duration enhances or sustains performance on sensory and motor tasks, but impairs executive control processes.

Although previous empirical work and reviews have advanced our understanding of exercise-cognition relationships, many questions still remain. Given the complexities in both cognitive functioning and the physiological processes involved in physical exercise, it is unsurprising that the literature in this field varies greatly. Moreover, more than 30 studies have been published since the last comprehensive review of this field. We believe an updated discussion of the critical characteristics of this literature, as well as specific avenues for future research, may promote advancements both by those working within this field, or others new to this area. Therefore, in this review we aim to answer the following questions: (1) what has been done? (2) what has been found? (3) what is next?

## What Has Been Done?

### Gathering Extant Literature

In this review we will explore extant research examining cognitive changes during acute exercise (see Pontifex et al., 2019 for review of cognitive changes following acute exercise). More specifically, this review is limited to studies that employed a bout of aerobic exercise and collected measures of cognitive performance while the participant was in the process of engaging in physical activity. Additionally, we restricted inclusion to studies manipulating exercise load using intensity or duration and excluded studies using physical load manipulations (i.e., load carriage). The review is limited to studies using healthy young adult populations. To identify studies eligible for this review, a computerized search for studies in Google Scholar and Pubmed (through May 30, 2021) was conducted using the following keywords: searches used the logical operator “OR” between exercise-related terms (i.e., “exercise,” “physical exercise,” “physical activity”) and the logical operator “AND” between the exercise-related terms and either the cognition search modifier cogniti* (i.e., “cognition,” “cognitive”), or cognitive tasks previously identified in reviews of this field (i.e., “stroop task”) (see Pontifex et al., 2019 for list of cognitive tasks). Unpublished and non-peer reviewed studies were excluded. This search strategy resulted in a total of 73 studies that investigated cognition during acute exercise. Studies were then classified by authors (JC and GG) with regards to sample size, characteristics of subject population, exercise intensity and duration, executive and non-executive cognitive domain and cognitive task, main findings (categorization provided in Tables 1–7). Study design, participant fitness level, exercise modality and specific timing of cognitive testing during exercise are provided in Supplementary Table 1. To note, all studies that met inclusion criteria used exercise modes of cycling (80% of studies), running or walking (20% of studies), with one study using kayak ergometry (Davranche et al., 2009).

Studies were also characterized with regards to the intensity and duration of the exercise bout (see Table 8 for classification criteria). Exercise intensity relates to how much energy is expended, or how hard your body is working during exercise. Common methods for prescribing and monitoring intensity during aerobic exercise include percentage of maximum heart rate (HRmax), heart rate reserve (HRR), maximal oxygen uptake (V0_2_max), or oxygen uptake reserve (VO_2_reserve). Studies using these methods to prescribe relative intensity (i.e., energy cost in relation to an individual’s max capacity) were then classified into intensity categories using criteria from American College of Sports Medicine guidelines for Exercise Testing and Prescription (Table 6.1; Garber et al., 2011). Intensity categories included very-light (i.e., < 37% VO_2_max or < 57% HRmax), light (37–45% VO_2_max or 57–63% HRmax), moderate (45–63% VO_2_max or 64–76% HRmax), vigorous (64–90% VO_2_max or 77–95_%_ HRmax) and near-maximal to maximal (≥ 91% VO_2_max or ≥96% HRmax). If maximum power output (*W*max) values were reported, conversion formulas were used to obtain %V0_2_max (Arts and Kuipers, 1994). Additionally, we explored whether these effects were moderated by factors such as characteristics of the participants (i.e., fitness level, sports expertise), exercise protocol (intensity, duration, mode), and dependent outcome measure (response time, accuracy).

**TABLE 1:** Summary of studies examining inhibition during acute aerobic exercise.

Sample characteristics				Exercise			Main results		
Study (year)	*n* (F)	Age	Prescribed intensity	Intensity category	Duration^a^	Cognitive task	Response time	Accuracy	Did performance improve?
John et al. (2009)	20 (9)	26.4 ± 4.04 years	Rest, 1 MPH	Rest, very light	NR	Stroop	No differences	No differences	No effects
Davranche et al. (2009)	14 (3)	30 ± 8 years	Rest, 50% MAP	Rest, moderate	2 15-min Periods (5 min rest between)	Eriksen flanker	Moderate < rest	No differences	Yes; only RT
Ando et al. (2011)	12 (0)	25.3 ± 3.1 years	Rest, 40, 60, 80% VO_2_peak	Rest, light, moderate, vigorous	6.5 min	Eriksen flanker	Overall RT (motor time): No differences; pre-motor time: moderate < rest	Error rate: rest < vigorous	No; ACC at vigorous
Labelle et al. (2013)	37 (18)	23.8 ± 2.6 years	40, 60, 80% PPO	Light, moderate, vigorous	6.5 min	Modified stroop	No differences	No differences	No effects
Pontifex and Hillman (2007)	41 (26)	20.2 ± 1.6 years	Rest, 60% HRmax	Rest, light	6.5 min	Eriksen flanker	No differences	Incongruent accuracy: light < rest; congruent accuracy: no differences	No; only ACC
Komiyama et al. (2019)	17 (0)	22.1 ± 1.7 years	Rest, 50%, 80% VO_2_peak (performed within 1 exercise session)	Rest, moderate, vigorous	8 min	Go/No-Go	No differences	Accuracy score: vigorous < rest	No; only ACC at vigorous
Ando et al. (2014)	12 (0)	22.9 ± 1.5 years	Rest, 60% VO_2_peak	Rest, moderate	10 min	Go/No-Go	Moderate < rest	No differences	Yes; only RT
Smith et al. (2016)	15 (9)	28 ± 5 years	Rest, 70, 90% HRR	Rest, vigorous, near-maximal	10 min	Go/No-Go	Near-maximal > vigorous, rest	Omission errors: vigorous, rest < near-maximal; false alarms: vigorous, rest < near-maximal	No; RT and ACC at near-maximal
Ando et al. (2013)	12 (0)	22.9 ± 1.5 years	Rest, 60% V0_2_peak (∼80% HRmax)	Rest, vigorous	10 min (5 min warm-up)	Go/No-Go	Vigorous < rest	No differences	Yes, only RT
Davranche et al. (2009)	12 (4)	18 ± 4 years	40 ± 4, 75 ± 2% HRmax	Very light, moderate	14 Min (10 min warm-up)	Simon	Moderate < very light	No differences	Yes; only RT at higher intensity
Davranche et al. (2015)	14 (3)	21 ± 2 years	VT—20%, VT, VT + 20% (∼74, 81, 90% HRmax)	Moderate, vigorous	15 min (5 min warm-up)	Simon	Min 10 moderate, vigorous < min 0 moderate, vigorous	No differences	Yes; only RT
McMorris et al. (2009)	24 (0)	24.32 ± 7.10 years	Rest, 50, 80% MAP	Rest, moderate, vigorous	15 min (5 min warm-up)	Eriksen flanker	Overall RT: moderate, rest < vigorous; RT post-correct responses: moderate, rest < vigorous; RT post-error responses: no differences	Errors: moderate, rest < vigorous	No; RT and ACC at vigorous
Komiyama et al. (2017)	13 (0)	21.5 ± 3.5 years	Rest, 50% VO_2_peak	Rest, moderate	15 min (5 min warm-up)	Go/No-Go	Light < Rest	No differences	Yes; only RT
Schmit et al. (2015)	15 (5)	22.1 ± 0.6 years	Rest, 80% MAP	Rest, vigorous	20 min (3 min warm-up)	Eriksen flanker	No differences	Incongruent errors: rest < vigorous; congruent errors: no differences	No; only ACC
Finkenzeller et al. (2019)	52 (24)	22.12 ± 2.56 years	Rest, 30, 30 + 60, 60% MAP	Rest, very light, very light/moderate varied, moderate	20 min (4 min warm-up)	Eriksen flanker	Congruent RT: Min 16 Very light/moderate < rest; Min 8, 12, 16 moderate < rest	Incongruent errors: moderate > rest; congruent errors: no differences	Yes; RT No; ACC
Davranche and McMorris (2009)	12 (4)	32 ± 9 years	Rest, lactate threshold power (77 ± 4% HRmax)	Rest, moderate	21 Min	Simon	Overall RT: moderate < rest; simon effect: rest < moderate	No differences	No; only RT
Joyce et al. (2009)	10 (3)	23 ± 2 years	Rest, 40% MAP	Rest, light	25 min (4 min warm-up)	Stop-signal	Go RT: light < rest; stop-signal RT: light < rest	No differences	Yes; only RT
Huertas et al. (2011)	30 (0)	17 ± 2 years	Rest, 80, 95% LT	Rest, moderate, vigorous	25 min (10 min warm-up)	ANT-I	No differences	No differences	No effects
Olson et al. (2016)	27 (11)	20.4 ± 2.0 years	Rest, 40, 60% VO_2_peak	Rest, light, moderate	26 min (5 min warm-up)	Eriksen flanker	Moderate < light, rest	Incongruent accuracy: light, moderate < rest; congruent accuracy: no differences	Yes; RT At moderate: No; ACC
Komiyama et al. (2015)	16 (0)	23.0 ± 2.3 years	140 bpm	Rest, moderate	30 min (5 min warm-up)	Go/No-Go	Min 23 moderate < rest	No differences	Yes; only RT
Komiyama et al. (2016)	10 (0)	22.3 ± 2.1 years	Rest, 140 bpm (∼71% HRmax)	Rest, moderate	30 min (5 min warm-up)	Go/No-Go	Moderate < rest	Go accuracy: rest < moderate; NoGo accuracy: no differences	Yes; RT and ACC
Sanchis et al. (2020)	24 (0)	22.6 ± 2.9 years	80% VT1, 80% VT2	Light, moderate	33 min 45 s (6.5 min warm-up)	ANTI-I (Executive)	No differences	No differences	No effects
Tempest et al. (2017)	14 (5)	22.7 ± 3.8 years	<30 Watts, 10% Above VT	Very light, vigorous	60 min	Eriksen flanker	RT: end-vigorous exercise < beginning-vigorous exercise	Incongruent accuracy: end- very light, end-vigorous < beginning-very light, beginning vigorous; congruent accuracy: no differences	Yes; RT at higher intensity No; ACC
Giles et al. (2018)	36 (21)	23.4 ± 3.6 years	57, 70% HRmax	Light, moderate	90 min (5 min warm-up)	Stroop	Min 30 moderate < Min 30 light; Min 90 moderate < Min 90 light	No differences	Yes; only RT at higher intensity

*n, number of participants; F, females; “-” = not measured; RT, reaction time; ACC, accuracy; NR, not reported; Simon Effect = Incongruent RT-Congruent RT; Accuracy Score = Correct Responses/Total Responses; ANT-I, attentional network task (inhibition); VO_2_max, maximum volume of oxygen uptake; VO_2_peak, peak oxygen uptake; HRmax, maximum heart rate; HRR, heart rate reserve; VT, ventilatory threshold; PPO, peak power output; MAP, maximum aerobic power; VAT, ventilatory anaerobic threshold; RPE, rating of perceived exertion.*

*^a^Exercise duration excludes warm-up time.*

**TABLE 2:** Summary of studies examining working memory during acute aerobic exercise.

Sample characteristics				Exercise			Main results		
Study (year)	*n* (F)	Age	Prescribed intensity	Intensity category	Duration^a^	Cognitive task	Response time	Accuracy	Did performance improve?
Quelhas Martins et al. (2013) Experiment 2	55 (65)	19.57 ± 0.83 years	Rest (within) and 5 watts, 50–60 watts, or 75–90 watts (between) (41, 61 and 64% HRmax)	Rest, very light, light and moderate	NR: ∼20 min (2 min warm-up)	Sternberg	Response latency: light, moderate < rest	-	Yes
Komiyama et al. (2019)	17 (0)	22.1 ± 1.7 years	Rest, 50, 80% VO_2_peak (performed within 1 exercise session)	Rest, moderate, vigorous	8 min	Spatial DR	No differences	Accuracy: vigorous < rest	No; only ACC
Komiyama et al. (2017)	13 (0)	21.5 ± 3.5 years	Rest, 50% V0_2_peak	Rest, moderate	15 min (5 min warm-up)	Spatial DR	No differences	No differences	No effects
Dutke et al. (2014)	60 (14)	M = 26.1 years	75, 120% VT (∼53–73, 76–95% HRmax)	Light-moderate, vigorous	∼27 min (10 min warm-up)	Word comparison task (with button press every 2 s)	No differences	No differences	No effects
Komiyama et al. (2016)	10 (0)	22.3 ± 2.1 years	Rest, 140 bpm (∼71% HRmax)	Rest, moderate	30 min (5 min warm-up)	Spatial DR	No differences	No differences	No effects
Komiyama et al. (2015)	16 (0)	23.0 ± 2.3 years	140 bpm (∼ 71% HRmax)	Rest, moderate	30 min (5 min warm-up)	Spatial DR	No differences	No differences	No effects
Travlos and Marisi (1995)	20 (0);10 low fit, 10 high fit	23.3 ± 3.8 years	40, 50, 60, 70, 80% VO_2_max	Light, moderate, vigorous,	60 min	RNG	-	No differences	No effects
Rattray and Smee (2016)	20 (10)	Males: 26.6 ± 5.2; Females: 24.6 ± 5.6 years	Rest, 90% VT (moderate constant), 40 min at 90% VT then two 3-min intervals at 90 then 50% V0_2_max (moderate varied), 40 min at 90% VT then two 3-min intervals at 50 then 90% VO_2_max (moderate-vigorous varied)	Rest, moderate constant, moderate varied, moderate-vigorous varied	60 min	Speed match	RT: moderate constant, moderate-vigorous varied < rest	No differences	Yes, only RT
Tempest et al. (2017)	14 (5)	22.7 ± 3.8 years	< 30 watts; 10% above VT	Very light, vigorous	60 min	2-Back Task	-	*d*’: end-vigorous < beginning-vigorous	No, only ACC at higher intensity

*n, number of participants; F, females; M, mean; “-” = not measured; RT, reaction time; ACC, accuracy; NR, not reported; DR, delayed response task; RNG, Random Number Generation; VO_2_max, maximum volume of oxygen uptake; V0_2_peak, peak oxygen uptake; HRmax, maximum heart rate VT, ventilatory threshold.*

*^a^Exercise duration excludes warm-up time.*

**TABLE 3:** Summary of studies examining cognitive flexibility during acute aerobic exercise.

Sample characteristic				Exercise			Main results		
Study (year)	*n* (F)	Age	Prescribed intensity	Intensity category	Duration^a^	Cognitive task	Response time	Accuracy	Did performance improve?
Oppezzo and Schwartz (2014) Experiment 1	48 (NR)	NR	Rest, self-selected walking at “comfortable” pace	Rest, very light-light	NR	GAU; CRA	–	GAU creative appropriate uses: rest < very light-light; CRA number of correct responses: very light-light < rest	Yes; no
Oppezzo and Schwartz (2014) Experiment 2	48 (NR)^b^	NR	Rest, self-selected walking at “comfortable” pace	Rest, very light- light	NR	GAU	–	Creative appropriate uses: rest < very light-light	Yes
Pesce et al. (2003)	16 (8)	19–40 years	Rest, 60% V0_2_max	Rest, moderate	NR	Local global task with switching condition	Overall RT: moderate < rest; switch cost: moderate < rest	No differences	Yes; only RT
Pesce et al. (2007) Experiment 1	24 (0) Elite soccer players, 24 (0) Physically active controls	17.9 ± 0.8 years	Rest, 60% HRR	Rest, vigorous	NR	Local global task with switching condition	Overall RT: non-athletes: vigorous < rest; athletes: no differences; switch cost: non-athletes: vigorous < rest, athletes: no differences	–	Yes; only RT in non-athletes
Pesce et al. (2007) Experiment 2	Same participants and design as Exp 1		Rest, 60% HRR	Rest, vigorous	NR	Local global task with switching condition	Overall RT: vigorous < rest; RT switch cost: athletes: vigorous < rest, non-athletes: no differences	-	Yes; only RT in athletes
Labelle et al. (2013)	37 (18)	23.8 ± 2.6 years	40, 60, 80% PPO (performed within 1 exercise session)	Light, moderate, vigorous	6.5 min	Modified Stroop with switching condition	No differences	Switching error rate: light, moderate < vigorous; reading non-switch error rate: light, moderate < vigorous; inhibition non-switch error rate: no differences	No; only ACC at vigorous
Del Giorno et al. (2010)	30 (13)	20.2 ± 1.1 years	Rest, 75% VT, VT	Rest, light, vigorous	25 min (5-min warm-up)	WCST	-	Unique errors: rest < light, vigorous; perseverative errors: no differences; total number of errors: no differences	No
Wang et al. (2013)	80 (31)	20.51 ± 1.99 years	Rest, 30, 50, 80% HRR	Rest, light, moderate, vigorous	∼30 min (6 min warm-up)	WCST	-	Perseverative errors: moderate, light, rest < vigorous; correct conceptual-level responses: vigorous < moderate, light, rest; number of categories completed: vigorous < moderate, light, rest; failure to maintain set: no differences	No; only ACC at vigorous
Dietrich and Sparling (2004)	24 (0)^b^	23.7 ± 9.4 years	Rest, 70–80% HRmax (∼140–160 bpm) running or cycling	Rest, moderate-vigorous	45 min (5-min warm-up)	WCST-64	-	Correct conceptual-level responses: moderate-vigorous < rest; total number of errors: rest < moderate-vigorous; perseverative reponses (total): rest < moderate-vigorous: perseverative errors: no differences	No

*n, number of participants; F, females; “−” = not measured; RT, reaction time; ACC, accuracy; NR, not reported; GAU, Guilford’s alternate uses test; CRA, compound remote-association test; WCST, Wisconsin Card Sorting Task; WCST-64, Wisconsin Card Sorting Task (shortened version); VO_2_max, maximum volume of oxygen uptake; V0_2_peak, peak oxygen uptake; HRmax, maximum heart rate VT, ventilatory threshold; MET, metabolic equivalent expenditure; PPO, peak power output; Switch Cost = RT difference switching between global to local trials or vice versa.*

*^a^Exercise duration excludes warm-up time.*

*^b^Additional sample characteristics provided in Supplementary Table 1.*

**TABLE 4:** Summary of studies examining attention during acute aerobic exercise.

Sample characteristics				Exercise			Main results		
Study (year)	*n* (F)	Age	Prescribed intensity	Intensity category	Duration^a^	Cognitive task	Response time	Accuracy	Did performance improve?
Pesce et al. (2002) Experiment 1	16 (8)	19–40 years	Rest, 60% V0_2_max	Rest, moderate	NR	Local global task	Overall RT: moderate < rest; RT during exercise: global targets < local targets	No differences	Yes; only RT
Pesce et al. (2002) Experiment 2	Same participants and design as Exp 1		Rest, 60% V0_2_max	Rest, moderate	NR	Local global task	Moderate < rest	No differences	Yes; only RT
McMorris and Graydon (1997) Experiment 1	12 (0) College soccer players	20.8 ± 1.34 years	Rest, 70, 100% MAP	Rest, moderate, near-maximal	NR	Visual search in game simulations	Speed of search (RT): near-maximal < moderate, rest	No differences	Yes; only RT near-maximal
Pesce et al. (2004) Experiment 1	42 (20)	Males: 21.9 ± 4.2; Females: 22.5 ± 4.3 years	Rest, 60% HRR	Rest, vigorous	NR: ∼6–8 min (2 min warm-up)	Local global task	RT: Vigorous < Rest	No differences	Yes; only RT
Pesce et al. (2004) Experiment 2	Same participants and design as Exp 1		Rest, 60% HRR	Rest, vigorous	NR: ∼6–8 min (2 min warm-up)	Local global task	Overall RT: Vigorous < rest; RT during Exercise: Males < Females	No differences	Yes; only RT
Quelhas Martins et al. (2013) Experiment 1	24 (0): Exercise 12; Control 12	20.50 ± 0.89 years	Rest, 60–77% HRmax (ranged from 60 to 180 watts across 4 blocks)	Rest, moderate-vigorous	NR: ∼8 min	PASAT	-	Percentage of correct responses: rest < moderate–vigorous	Yes
González-Fernández et al. (2017) Experiment 1	24 (12)	20.29 ± 0.95 years	40, 60, 80, 100% of VAT (<50, 50–64, 64–77, 85–100% HRmax)	Very light, light, moderate, vigorous-maximal	5 min (3 min warm-up)	PVT	RT: very light, light, moderate < vigorous-maximal	-	Yes; only RT at lower intensity
Wohlwend et al. (2017)	15 (15)	24.3 ± 3.3 years	40, 60% VO_2_max	Light, moderate	5 min (5 min warm-up)	CPT	No differences	Accuracy: moderate < light	No; only ACC at higher intensity
Yagi et al. (1999)	24 (12)	Females: 20.6 ± 2.5; Males: 19.9 ± 1.7 years	Rest, 130–150 bpm	Rest, moderate	10 min	Visual and auditory oddball tasks	Overall RT: moderate < rest: RT during exercise: visual task < auditory task	Accuracy: moderate < rest	Yes
Ciria et al. (2019)	20 (0) Cyclists	M = 23.9 years	30, 80% VO_2_max	Very light, vigorous	20 min (10 min warm-up)	Visual oddball task	No differences	No differences	No effects
Sanabria et al. (2011)	22 (2)	18–22 years; M = 22	Active rest/very light (cycling with no resistance), 85% anaerobic threshold	Very light, moderate-vigorous	20 min	Posner spatial cueing task	RT: moderate-vigorous < very light	-	
Del Giorno et al. (2010)	31 (13)	20.2 ± 1.1 years	Rest, 75% VT, VT	Rest, moderate, vigorous	25 min (5-min warm-up)	CPT	-	False alarms: rest < moderate < vigorous	No; ACC worse at higher intensity
Huertas et al. (2011)	30 (0)	17 ± 2 years	Rest, 80, 90% LT (∼75 ± 3, 86 ± 3% HRmax)	Rest, moderate, vigorous	25 min (10 min warm-up)	ANT	Overall RT: vigorous < rest; alerting RT: moderate < rest	No differences	Yes; only RT
Hüttermann and Memmert (2014)	8 (4) Non-athletes; 8 (2) Team sports athletes	Overall: 25.47 ± 3.76 years; Non-athletes: 26.00 ± 4.27; Athletes: 24.88 ± 5.72	50, 60, 70% HRmax (performed within 1 exercise session)	Very light, light, moderate	30 (5 min warm-up)	Attentional breadth task	-	Success rate: non-athletes across all exercise conditions < athletes; athletes: very light < light < moderate; non-athletes: moderate < very light, light	Yes; ACC in athletes No; ACC at higher intensity in non-athletes
Sanchis et al. (2020)	24 (0)	22.6 ± 2.9 years	80% VT1, 80% VT2	Light, moderate	33 min 45 s (6.5 min warm-up)	ANTI-I (Arousal)	ANT-I overall RT: moderate < light; AV RT: no differences	No differences	Yes; only RT at moderate
Lambourne et al. (2010)	19 (11)	21.37 ± 0.9 years	Rest, 90% VT (mean HR = 143 ± 13 bpm; RPE = 13 ± 1)	Rest, moderate	35 min (5-min warm-up)	PASAT	-	No differences	No effects
Radel et al. (2018)	12 (0) Trained cyclists	27.8 ± 2.0 years	Rest, 50 watts, VT, VT ± 15% (moderate-varied)	Rest, light, moderate, moderate-varied	40 min (10 min warm-up)	SART	Go RT: moderate, moderate-varied < rest, light; moderate-varied < moderate	False alarms: rest < light, moderate, moderate-varied	Yes; RT at moderate No; ACC at light to moderate
González-Fernández et al. (2017) Experiment 2	18 (18)	19.94 ± 1.98 years	Low effort, 75% VAT (∼44, 63% HRmax)	Very light, light	45 min (3 min-warm up)	PVT	Light < very light	-	Yes; only RT at light
Bullock et al. (2015)	12 (6)	20 ± 1.08 years	Rest, 7–9, 12–14 RPE	Rest, light, moderate	45 min (5-min warm-up)	Visual oddball task	moderate < rest, light	No differences	Yes; only RT in moderate

*Note. n, number of participants; F, females; “-” = not measured; RT, reaction time; ACC, accuracy; NR, not reported; PASAT, Paced Auditory Serial Addition Task; PVT, psychomotor vigilance task; CPT, continuous performance task; ANT, attentional network task; SART, Sustained Attention to Response Task; VO_2_max, maximum volume of oxygen uptake; V0_2_peak, peak oxygen uptake; HRmax, maximum heart rate; HRR, heart rate reserve; VT, ventilatory threshold; MAP, maximum aerobic power; VAT, ventilatory anaerobic threshold; RPE, rating of perceived exertion.*

*^a^Exercise duration excludes warm-up time.*

**TABLE 5:** Summary of studies examining motor speed during acute aerobic exercise.

Sample characteristics				Exercise			Main results		
Study (year)	*n* (F)	Age	Prescribed intensity	Intensity category	Duration^a^	Cognitive task	Response time	Accuracy	Did performance improve?
John et al. (2009)	20 (9)	26.4 ± 4.04 years	Rest, 1 MPH	Rest, very light	NR	Typing, mouse proficiency	Typing speed: very light < rest	Mouse proficiency: moderate < rest	Yes
Arcelin et al. (1998)	22 (12)	23.5 ± 4.3 years	Rest, 60% MAP	Rest, moderate	3 10-min bouts	CRT	Moderate < rest	Error rate: moderate < rest	Yes
Chmura et al. (1998)	17 (0)	Above LT: 24.8 ± 1.4; Below LT: 22.6 ± 1.8 years	70% Below LT, 10% Above LT	Very light, moderate	Above LT: 20 min; Below LT: 60 min	CRT	Min 10–20 very light < pre-exercise; Min 10–60 moderate < pre-exercise	-	Yes
Ando et al. (2010)	10 (0)	25.1 ± 3.4 years	Rest, 40, 60, 80% VO_2_peak	Rest, light, moderate, vigorous	6.5 min	SRT to peripheral visual stimuli	Overall RT: no differences; premotor time: rest < vigorous	No differences	No, only in vigorous
Ando et al. (2008)	12 (0)	26.2 ± 3.1 years	Rest, 65% VO_2_peak	Rest, vigorous	10 min	SRT	Overall RT: no differences; peripheral visual premotor time: rest < vigorous; central visual premotor time: no differences	No differences	No
Arcelin and Brisswalter (1999)	19 (9)	23.7 ± 3.3 years	Rest, 60% MAP	Rest, moderate	10 min	CRT	No differences	No differences	No
Brisswalter et al. (1997)	20 (0)	Trained: 23.3 ± 1.5; untrained: 23.7 ± 1.8 years	Rest, 20, 40, 60, 80% MAP	Rest, very light, light, moderate, vigorous	10 min	SRT	RT untrained: light, moderate, vigorous > rest; RT trained: light > rest	No differences	No
Davranche and Audiffren (2004)	16 (7)	22.8 ± 2.5 years	Rest, 20, 50% MAP	Rest, very light, moderate	17 min	CRT	RT: moderate < rest, very light, rest	No differences	Yes, only in moderate
Paas and Adam (1991)	16 (4)	26.6 ± 5.6 years	Rest, 5, 40/85, 75% Wmax	Rest, very light, moderate-vigorous, vigorous	20 min (10 min warm-up)	CRT	RT: very light, moderate-vigorous, vigorous < rest	Error rate: very light, moderate-vigorous, vigorous < rest	Yes
Audiffren et al. (2008)	17 (8)	Women: 21.13 ± 1.13, Men: 22.00 ± 1.22 years	Rest, 90% VT	Rest, moderate	35 min	CRT	RT: Min 14–39 moderate < Min 14–39 rest	-	Yes
Collardeau et al. (2001)	11 (NR)	26.5 ± 4.8 years	Rest, 100% VT	Rest, vigorous	90 min	SRT	Min 40 vigorous < pre-vigorous	No differences	Yes
Collardeau et al. (2001)	8 (NR)	24.3 ± 3.4 years	Rest, 100% VT	Rest, vigorous	100 min	SRT	RT: pre-vigorous < vigorous	No differences	No

*n = number of participants; F = females; “-” = not measured; RT = reaction time; NR = not reported; CRT = Choice Reaction Time Task; SRT = Simple Reaction Time Task; MAP = maximum aerobic power; VO2max = maximum volume of oxygen uptake; V0_2_peak = peak oxygen uptake; Wmax = maximum power output; HRmax = maximum heart rate; VT = ventilatory threshold; LT = lactate threshold.*

*^a^Exercise duration excludes warm-up time.*

**TABLE 6:** Summary of studies examining information processing during acute aerobic exercise.

Sample characteristics				Exercise			Main results		
Study (year)	*n* (F)	Age	Prescribed intensity	Intensity category	Duration^a^	Cognitive task	Response time	Accuracy	Did performance improve?
McMorris and Graydon (1997) Experiment 1	12 (0)	20.8 ± 1.34 years	Rest, 70, 100% MAP	Rest, vigorous, near-maximal	NR	Visual search in game simulations	Near-maximal < vigorous, rest	-	Yes; only RT in near-max
McMorris and Graydon (1997) Experiment 2	12 (0)	20.8 ± 1.78 years	Rest, 70, 100% MAP	Rest, vigorous, near-maximal	NR	Soccer decision-making/problem solving	Total speed of decision: vigorous, near-maximal < rest; speed of decision following ball detection: near-maximal < rest, vigorous	Accuracy: rest < near-maximal	Yes
Adam et al. (1997)	20 (9)	26.4 ± 5.1 years	5, 75% Wmax	Very light, vigorous	20 min (10 min warm-up)	SIT decision task; STM decision task	Vigorous < very light	No differences	Yes; only RT in higher intensity
Paas and Adam (1991)	16 (4)	26.6 ± 5.6 years	Rest, 5, 40/85, 75% Wmax	Rest, very light, moderate-vigorous, vigorous	20 min (10 min warm-up)	Backward masking task	-	Letters correct: during exercise < before, after exercise	No
Shields et al. (2011)	33 (17)	Women: 20.7 ± 1.9; Men: 23.1 ± 3.5 years	Rest, 45, 80% HRmax	Rest, very light, vigorous	20 min	Visual threat detection	Very light, vigorous < rest	Overall accuracy: rest < very light, vigorous; discrepant fear-irrelevant accuracy: vigorous < very light	Yes
Lambourne et al. (2010)	19 (11)	21.1 ± 1.7 years	Rest, 90% VT (mean HR = 143 ± 13 bpm; RPE = 13 ± 1)	Rest, moderate	40 min	CFF	-	CFF score: Min 28, Min 30 rest < Min 28, Min 30 moderate	Yes
Grego et al. (2004)	16 (0)	Endurance-trained: 30.8 ± 7.3; regular trained: 29.4 ± 4.8 years	Rest, 60% VO_2_max	Rest, moderate	180 min	CFF	-	CFF mdi: Min 120 moderate < Min 20 moderate; CFF mtot: no differences	No

*n, number of participants; F, females; “-” = not measured; RT, reaction time; NR, not reported; SIT, sustained information transfer; STM, short term memory; CFF, critical flicker fusion; MAP, maximum aerobic power; VO_2_max, maximum volume of oxygen uptake; V0_2_peak, peak oxygen uptake; Wmax, maximum power output; HRmax, maximum heart rate; VT, ventilatory threshold; RPE, rating of perceived exertion; CFF, critical flicker fusion; CFF mtot, total mean of ascending and descending values; CFFmdi, mean of difference between ascending and descending values.*

*^a^Exercise duration excludes warm-up time.*

**TABLE 7:** Summary of studies examining memory during acute aerobic exercise.

											
Sample characteristics				Exercise		Stage of memory			Main results		
Study (year)	*n* (F)	Age	Prescribed intensity	Intensity category	Duration^a^	Start encoding^b^	Start retrieval	Cognitive task	Response time	Accuracy	Did performance improve?
											
Pyke et al. (2020) Experiment 3	23 (20)	19.62 ± 1.51 years	High-intensity interval training (HIIT), 65–75% HRmax	Moderate, vigorous	6 min	Immediately pre-exercise	90 min post-encoding	Old/New recognition task	-	Moderate > passive rest	Yes
Keyan and Bryant (2017b)	49 (33)	Exercise: 19.96 ± 2.32; Walking: 19.79 ± 2.70 years	Walking, 50–85% HRmax	Very light-vigorous	10 min	Immediately pre-exercise	2 days post-exercise	Cued recall	-	Cued recall: no differences; Intrusive Memories: walk < exercise	Yes
Miles and Hardman (1998)	24 (18)	M = 20.3 years	120–150 bpm	Very light-moderate	11 min	During exercise, rest	During exercise, rest	Word list recall	-	Correct Free recall: exercise-rest, rest-exercise < rest-rest, exercise-exercise; false alarms: no differences	No effects
Crawford et al. (2021) Experiment 2	68 (38)	20.79 ± 1.98 years	80% HRR	Vigorous	15 min	Immediately pre-exercise (List 1), 5 min after (List 2)	Immediately pre-exercise (list 1), 5 min after (List 2)	AB/AC memory interference task	-	Memory interference: vigorous > rest	No
Frith et al. (2017)	88 (48)	21.9 ± 2.4 years	Rest, self-selected	Rest, light to near-maximal	15 min	Immediately before, min NR during, min 5 AFTER	Min 20, Hr 24 post-exercise	RAVLT; prospective memory task	-	Short-term memory: no differences; learning: no differences; 20-min long-term memory: exercise during encoding < exercise pre-encoding, rest; 24-h long-term memory: no differences; 24-h attribution memory: exercise during encoding < exercise pre-encoding; prospective memory: no differences	Yes
Loprinzi et al. (2021)	150 (88)	Exercise: 20.32 ± 1.3; Control: 20.17 ± 1.3 years	80% HRR	Vigorous	20 min	Incidental encoding immediately pre-exercise, intentional encoding immediately post-exercise	0, 30 min post-exercise	Incidental memory processing task, incidental encoding task	-	No differences	No effects
Loprinzi et al. (2021) Experiment 1	47 (27)	21.1. ± 1.7 years	75% HRR	Vigorous	20 min	5 min post-exercise	55 min, 24 h post-encoding	Word list task	-	Exercise > rest through encoding, retrieval	Yes
Loprinzi et al. (2021) Experiment 2	42 (23)	20.6 ± 1.1 years	75% HRR	Vigorous	20 min	Immediately pre-exercise	4 h, 20 min, 24 h post-encoding	Word list task	-	Exercise > rest through consolidation	Yes
Loprinzi et al. (2021) Experiment 3	31 (27)	20.5 ± 1.0 years	75% HRR	Vigorous	20 min	2 h pre-exercise	4 h, 24 h post-encoding	Word list task	-	No differences	No effects
Silvers et al. (2018)	72 (49)	20.6 ± 1.9 years	Rest, 40, 60, 80% HRmax	Rest, very light, light, vigorous	20 min	During exercise, rest	Immediately, 1 Week Post-exercise	Multiple choice recall	-	No differences	No effects
											
Keyan and Bryant (2017a)	54 (26)	19.48 ± 3.03 years	60–70 rpm (76% MAP)	Vigorous	∼25 min	2 days pre-exercise [reactivity condition during exercise]	2 days post-exercise	Cued recall	-	Recall for central details: reactivation alone, vigorous alone < reactivation + vigorous; recall for peripheral details: no differences; intrusive memories: no differences	Yes
Hötting et al., 2016	81 (40)	22.00 ± 2.36 years	Rest, 57, 80% HRmax	Rest, very light-light, vigorous	30 min	10-min pre-exercise	20-min, 24-h post-exercise	Vocabulary test	-	Memory 20-min post-exercise (60-min post-encoding): No differences; memory 24-h post-exercise: rest < vigorous	Yes
Labban and Etnier (2011)	48 (33)	M = 22.02 years	RPE 13–15	Moderate-vigorous	30 min	Immediately pre-exercise, immediately post-exercise	35-min post-encoding	New York University paragraph recall test	-	Recall: encoding at rest < encoding post-exercise, encoding pre-exercise	Yes
Pyke et al. (2020) Experiment 1	19 (11)	21.85 ± 2.43 years	55–65, 65–75, 75–85% HRmax	Light, moderate, vigorous	30 min	Immediately pre-exercise	80 min post-encoding	Old/New recognition task	No differences	Moderate > vigorous	Yes
Wang et al. (2020)	22 (0)	21.6 ± 3.0 years	60–70% HRmax	Moderate	30 min	Immediately pre-exercise, immediately post-exercise	1, 24 h post-encoding	DM Task, SRTT, Procedural memory	SRTT RT Post-acquisition exercise < control	Words recalled: exercise pre-acquisition > post-acquisition, control at 1 h, pre-acquisition > control at 24 h; word recognition: exercise pre-acquisition, post-acquisition > control	Yes
van Dongen et al. (2016)	72 (48)	No exercise: 22.6 ± 2.8; immediate exercise: 21.5 ± 2.1; delayed exercise: 21.6 ± 2.4 years	80% HRmax	Vigorous	35 min	Immediately pre-exercise [Immediate], 4 h pre-exercise [Delayed]	2 days post-exercise	Cued recall	-	Recall: immediate exercise, no exercise < delayed exercise	Yes
Grego et al. (2004)	16 (0)	Endurance-trained: 30.8 ± 7.3; regular trained: 29.4 ± 4.8 years	60% VO_2_max	Moderate	180 min	Min 20, 40, 60, 80, 100, 120, 140, 160, 180 During, Min 5 After	Min 20, 40, 60, 80, 100, 120, 140, 160, 180 During, Min 5 After	Map recognition	Speed recognition: 80, 100, 120 min < 20 min; errors: 60, 80, 100 min < 20 min	Errors: Min 60, 80, 100 < Min 20	Yes

*Note. n, number of participants; F, females; “–”, not measured; RT, reaction time; AC, accuracy; NR, not reported; RAVLT, Rey Auditory Verbal Learning Test; DM, Declarative Memory Task; SRTT, Serial Reaction Time Task; VO_2_max, maximum volume of oxygen uptake; VO_2_peak, peak oxygen uptake; HRmax, maximum heart rate; HRR, heart rate reserve; VT, ventilatory threshold; PPO, peak power output; MAP = maximum aerobic power; VA, ventilatory anaerobic threshold; RPE, rating of perceived exertion.*

*^a^Exercise duration excludes warm-up time.*

*^b^Start of encoding must have taken place before or during exercise to be included.*

**TABLE 8:** Classification of aerobic exercise intensity.

	Relative intensity			
Intensity	%VO_2_max	%HRmax	%HRR or %VO_2_R	Perceived exertion (rating on 6–20 RPE scale)
Very light	<37	<57	<30	RPE < 9 (“Very light”)
Light	37–45	57–63	30–39	RPE 9–11 (“Very light to fairly light”)
Moderate	45–63	64–76	40–59	RPE 12–13 (“Fairly light to somewhat hard”)
Vigorous	64–90	77–95	60–89	RPE 14–17 (“Somewhat hard to very hard”)
Near-maximal to maximal	≥91	≥96	≥90	RPE ≥ 18 (“Very hard to maximal exertion”)

*Table adapted from Garber et al. (2011) and American College of Sports Medicine (2018). VO_2_peak = maximal oxygen uptake; %VO_2_max = percent of maximal oxygen uptake; HRmax, maximal HR; = %HRmax = percent of maximal HR; HRR, HR reserve; VO_2_R, oxygen uptake reserve; RPE, ratings of perceived exertion (Borg, 1982).*

### What Aspects of Cognition Have Been Investigated?

There are many ways one might characterize the types of cognitive functions that have been investigated during acute aerobic exercise. In this review, we classified the tasks used in each study by the executive or non-executive cognitive domain assessed (see Supplementary Table 1 for classification scheme). To date, executive function is the most extensively studied cognitive domain, comprising more than 45% of the total extant literature examining cognitive function during acute aerobic exercise. Therefore, we will primarily focus on the work that has been conducted within this domain, but will also briefly review other non-executive domains, including attention, motor speed, information processing, and memory.

#### Executive Functions

Executive functions, also referred to as cognitive control processes, are often grouped together as they all facilitate goal-directed behaviors and rely on a similar fronto–cingulo–parietal network (Alvarez and Emory, 2006). However, previous work has demonstrated that they are separable functions, including mental set shifting (moving back and forth between tasks, also termed cognitive flexibility), information updating (integrating new information, also termed working memory), and inhibition (withholding a prepotent response) (Miyake et al., 2000; Miyake and Friedman, 2012).

Although previous meta-analytic reviews have reported positive effect sizes when executive functions are measured during exercise, these conclusions are largely drawn from work examining inhibition (comprising 32% of all experiments measuring executive functions during exercise). As such, individual empirical studies examining single or separate executive functions have not consistently reported improved performance during exercise. Specifically, recent work has demonstrated that during exercise inhibitory control improves, working memory declines and effects on cognitive flexibility are dependent upon the specific task used to assess performance (Ando et al., 2014; Oppezzo and Schwartz, 2014). Thus, the effects on executive functions during exercise appear to be specific and dependent on several moderating variables (Dietrich and Audiffren, 2011; Audiffren, 2016). The domain-general effects often reported when cognition is measured following exercise may not necessarily be generalized across differing cognitive domains when cognition is measured during exercise. Therefore, here we will examine how separable executive functions of inhibition, working memory and cognitive flexibility have been studied and are impacted during exercise.

##### Inhibition

Inhibition (also referred to as “inhibitory control”) refers to the ability to suppress goal-irrelevant stimuli or behavioral responses. Inhibition can be dissociated into motor response inhibition and interference control (also termed cognitive inhibition) (Friedman and Miyake, 2004; Diamond, 2012; Nigg, 2017). Motor response inhibition involves inhibition of prepotent and automatic motor responses. Motor response inhibition has commonly been measured during exercise using non-selective stopping tasks, such as the Go No/Go Task (Donders, 1969) or Stop-Signal task (Logan and Cowan, 1984). Interference control refers to the ability to resist interference from goal-irrelevant stimuli within the environment. Interference control during exercise has been measured using stimulus-response compatibility tasks, such as the Stroop Test (Stroop, 1935), Eriksen Flanker Task (Eriksen and Eriksen, 1974), Simon Task (Simon and Wolf, 1963), or Attention Network Task (ANT)- Executive (Fan et al., 2002; see Table 1).

##### Working Memory

Working memory (also referred to as updating) refers to the ability to temporarily store and manipulate information (Miyake and Shah, 1999). Measures of working memory utilized during acute aerobic exercise include the n-back task, spatial delayed-response task (DR), Sternberg task, or random number generation task (RNG) (see Table 2).

##### Cognitive Flexibility

Cognitive flexibility (also referred to as “shifting”) refers to the ability to switch between different mental sets, tasks, or strategies (Miyake and Friedman, 2012). A wide range of tasks have been used to assess varying aspects of cognitive flexibility during exercise. Majority of studies have used the Wisconsin Card Sorting Task (WCST) to assess perseverance and set-shifting, or the local global task to assess switching between local and global attentional processing. Studies have also used Guilfords’ alternate uses task (GAU) and the compound remote associates task (CRA) to assess convergent and divergent thinking, and one study used a modified version of the Stroop task to assess task-switching (see Table 3).

#### Non-executive Functions

##### Attention

Moving beyond executive functions, research has also examined attention during exercise. Attention refers to the ability to selectively focus on relevant information while ignoring other perceivable information (Chun, 2000). Selective attention requires attending to relevant and ignoring non-relevant information, with common tasks used during exercise including odd-ball tasks (Herrmann and Knight, 2001) and local global tasks measuring focus of attention (Navon, 1977). Vigilance is the ability to sustain attention over time and measures used during exercise include continuous performance tasks (CPT), paced auditory serial addition tasks (PASAT), and psychomotor vigilance tasks (PVT) measuring sustained attention and vigilance (Gronwall, 1977; Dinges and Powell, 1985). Last, the Attention Network Task (ANT) has also been used to measure alerting (vigilance), orienting (selection), and executive control of attention (Fan et al., 2002; see Table 4).

##### Motor Speed

Motor speed includes several basic elements of motor activity, such as fine motor abilities (dexterity and speed) and reaction time (Harvey, 2019). Majority of studies have employed simple response time (SRT) and choice response time (CRT) tasks to evaluate reaction time during exercise (see Table 5). In a SRT there is a single stimulus and response type, whereas in a CRT there are multiple stimuli each requiring a different response type.

##### Information Processing

Information processing refers to the speed and accuracy of processing incoming information (Lichtenberger and Kaufman, 2009). A range of tasks have been used, such as Critical Flicker Fusion (CFF) and visual detection and visual search tasks (see Table 6).

##### Memory

Learning refers to a change in behavior resulting from experience, and memory refers to retaining and retrieving that information across the processes of encoding, storage, and retrieval (Crowder, 2015). The present review includes studies in which encoding occurred before or during exercise, such that at least one of three memory processes occurred during exercise. Memory tasks include cued, free or multiple choice recall tasks, prospective memory tasks and map recognition tasks (see Table 7). Given the various timing of when memory processes can be examined during exercise, this domain employs the widest variety of study designs, using both within and between-subjects designs.

## What Has Been Found?

Generally, the effects on cognitive performance during acute bouts of exercise are nuanced and dependent on the specific cognitive domain being assessed, as well as the intensity and duration of exercise. To add to the complexity of exercise-cognition interactions, effects of intensity and duration are often hard to disentangle due to their inherent physiological connections. However, intensity or duration of exercise may have differing implications and relevance depending on the particular population of interest or research question. Therefore, next we characterize findings by intensity and then by duration. Additionally, we outline research findings in terms of the behavioral dependent variables of response time and accuracy, since previous work has demonstrated that these variables may be differentially impacted by both intensity and duration (McMorris and Hale, 2012).

### Findings by Exercise Intensity

#### The Impact of Exercise Intensity on Executive Functions

##### Inhibition

Inhibition is the most extensively studied cognitive function within the field (comprising over 30% of the research on cognition during exercise and over 70% of the research on executive functions). More specifically, the majority of these studies examine inhibition at moderate intensity compared to rest. This breadth allows for a more in depth discussion of commonly observed trends, as well as mixed findings for this particular cognitive function. In general, acute exercise exerts variable effects on inhibitory control, yet certain trends have emerged which warrant further investigation (see Table 1). For example, exercise most often speeds response time for both interference control and motor response inhibition tasks, during moderate intensity, and exercise durations between 0 and 30 min. This beneficial effect of increased response time (without change in accuracy) has been widely observed in the literature and may be attributed to improved efficiency of peripheral motor processes during moderate exercise (Davranche et al., 2005, 2006). Another notable trend is that exercise most often reduces accuracy through increased error rates at increasing exercise intensities (moderate-to-vigorous) and during shorter durations (less than 20 min). This reduction in accuracy is primarily driven by false alarm rates, or incongruent errors, on trials requiring greatest amounts of executive control to engage in goal-directed behavior.

Below we examine the effects of specific exercise intensities compared to rest conditions (studies examining inhibition between exercise intensities are discussed in *section “Findings by Exercise Duration”*). We find that very light and light intensities exert variable effects on inhibitory control. Relative to rest, three studies demonstrated improved response times (Joyce et al., 2009; Komiyama et al., 2017; Finkenzeller et al., 2019) at very light and light intensity, and two demonstrated no effects (John et al., 2009; Ando et al., 2011). With regards to accuracy, two studies demonstrated decreased response accuracy for incongruent trials on the Eriksen Flanker task, with no changes to response time (Pontifex and Hillman, 2007; Olson et al., 2016). Taken together, very light and light intensity exercise may improve speed of motor response inhibition, but impair interference control. However, more research is needed since to date there are very few studies investigating these lower levels of exercise intensity.

Moderate intensity exercise consistently improves response time on inhibition tasks. In total, twelve studies have investigated motor response inhibition or interference control while participants either ran or cycled at moderate intensity, compared to a rest condition. Improvements in inhibitory control are primarily reported as improvements in response times across seven studies, with one study also demonstrating improved accuracy (Davranche et al., 2009; Ando et al., 2014; Komiyama et al., 2015, 2016, 2017; Olson et al., 2016; Finkenzeller et al., 2019). Three studies found decrements in inhibitory control at moderate intensity. Specifically, two found reductions in accuracy and one found slower response time when stimuli and responses are incompatible (Davranche and McMorris, 2009; Olson et al., 2016; Finkenzeller et al., 2019). In contrast, four studies reported no differences in either response times or accuracy, compared to rest (McMorris et al., 2009; Ando et al., 2011; Huertas et al., 2011; Komiyama et al., 2019). In sum, regardless of task type, improved response time is the most consistently observed finding impacting inhibition during moderate intensity when compared to rest. This is consistent with previous meta-analytic investigations demonstrating differing effects of acute exercise on response time vs. accuracy (McMorris and Hale, 2012).

Diving deeper, we find that a more consistent pattern emerges for tasks measuring motor response inhibition compared to those measuring aspects of interference control during moderate exercise. Specifically, relative to rest, response time improves on the Go-No/Go task during exercise, with no reported decrements in either response time or accuracy of performance (Ando et al., 2014; Komiyama et al., 2015, 2016, 2017, 2019). When compared to rest, accuracy does not appear to be significantly altered on less cognitively challenging non-selective stopping tasks, such as the GoNo/Go.

In contrast, when examining interference control at moderate intensity, results are more heterogeneous. Relative to rest, two studies reported overall improvements in response time regardless of stimulus-response compatibility (congruent or incongruent) (Davranche and McMorris, 2009; Davranche et al., 2009), whereas one reported improved speed for congruent trials and another reported slowed response times when switching from incongruent to congruent trials (Davranche and McMorris, 2009; Finkenzeller et al., 2019). Taken together, it appears that response time decreases to a similar extent on interference control and motor response inhibition tasks during acute moderate exercise. With regards to accuracy on interference tasks, one study found greater incongruent errors during acute moderate exercise (Olson et al., 2016), but negligible effects on response times or accuracy have also been reported (Ando et al., 2011; Huertas et al., 2011). In sum, decrements to performance on interference control tasks during moderate intensity stem from either slowed responding or reduced accuracy, specifically on incongruent trials. In contrast, ability to respond appropriately on congruent trials requiring less cognitive control appears to be unaltered. However, given that reported accuracy scores are often at ceiling (reported accuracy rates > 88%), the mixed findings with regards to accuracy of inhibitory control at moderate intensity may be due to failure to choose tasks which are complex enough to detect acute exercise-induced changes (McMorris and Hale, 2012).

Vigorous intensity has differing effects on response time and accuracy. Six studies investigated performance under vigorous intensity exercise compared to rest. Two studies demonstrated improved response times on Go/No-Go (Ando et al., 2013) or ANT tasks (Huertas et al., 2011 [marginal significance: *p* < 0.063]). In contrast, three studies demonstrated reductions in accuracy with no subsequent changes to response time (Ando et al., 2011; Schmit et al., 2015; Komiyama et al., 2019) and one study demonstrated decrements to both response time and accuracy (McMorris et al., 2009). Interestingly, similar to the decrements in performance reported at moderate intensity, these reductions in accuracy are specifically driven by No-Go or incongruent errors, where a participant fails to inhibit a prepotent response and/or responds to task-irrelevant aspects of the stimuli. No decrements were observed for congruent trials, suggesting that task conditions that elicit a higher level of conflict and require greater cognitive control may be more selectively influenced by acute exercise, and more so lead to impairments under higher levels of physical exertion. However, lack of differences in response time or accuracy between vigorous intensity and rest conditions have also been reported (Smith et al., 2016).

Increasing intensity to near-maximal may result in decrements to both response time and accuracy. To date, one study has demonstrated increased response times and reduced accuracy during maximal compared to both vigorous intensity and rest conditions (Smith et al., 2016). Interestingly, Schmit et al. (2015) observed a similar trend toward increased incongruent errors in the terminal period before exhaustion, suggesting that physiologically demanding conditions may impair multiple aspects of inhibitory control. However, further work is needed exploring cognitive performance under maximal effort before results can be generalized.

##### Working Memory

Very light and light intensities exert variable effects on working memory (see Table 2). For example, Quelhas Martins et al. (2013) found that response latency slopes were lower on a Sternberg task, indicating faster response times during light intensity cycling, whereas no differences were found during very light intensity, compared to rest. No effects on response times or accuracy between light, moderate and vigorous intensity conditions have also been reported (Travlos and Marisi, 1995).

Increasing intensity from very light-to-light to moderate may result in slight improvements or no changes in working memory. For example, compared to rest, two studies found improvements in response times, with no improvements in accuracy on Sternberg and speed match tasks (Quelhas Martins et al., 2013; Rattray and Smee, 2016). Furthermore, Rattray and Smee (2016) found that improved response times were demonstrated during moderate intensity conditions under both a constant or varied load. When measured using a spatial delayed response task, three studies found no differences in working memory during moderate vs. rest (Komiyama et al., 2016, 2017, 2019). Thus, across a variety of tasks, improvements or null effects have been reported.

Finally, of the three studies to assess vigorous intensity exercise, two found impairments in accuracy with no changes in response time compared to rest (Komiyama et al., 2019) and at the end vs. beginning of heavy exercise (Tempest et al., 2017), and one found no differences between light, moderate and vigorous intensities (Travlos and Marisi, 1995).

##### Cognitive Flexibility

Very light-to-light intensities may impair certain aspects of cognitive flexibility and enhance others (see Table 3). Very light intensity exercise differentially affects convergent and divergent thinking. For instance, in two experiments, Oppezzo and Schwartz (2014) measured performance on the GAU and CRA tasks during a self-selected “comfortable” walking condition, characterized as very-light to light intensity. Results demonstrated convergent thinking was impaired, with the number of correct responses generated during the CRA decreasing during exercise compared to rest (Experiment 2), whereas the number of creative, appropriate alternative uses generated during GAU increased (Experiments 1 and 2). Thus, convergent thinking may suffer, while divergent thinking may improve during bouts of light intensity exercise. However, intensity was not specifically determined using ACSM criteria and fitness level of participants and exercise duration were not reported, thus results should be interpreted with caution. More research is needed to understand how convergent and divergent thinking may be affected under varying exercise intensities. Additionally, more work is needed to understand how perseveration and set-shifting are affected during light intensity exercise. To date, two studies examined performance, finding an increase in the number of unique errors on the WCST during exercise compared to rest, or no differences between light intensity and rest (Del Giorno et al., 2010; Wang et al., 2013). Thus, light intensity may impair or enhance cognitive flexibility depending on the task used.

At moderate exercise intensity, cognitive flexibility remains relatively unaffected. For example, Pesce et al. (2003) found quicker response times when switching between local and global attending, with no changes in accuracy during exercise vs. rest. Labelle et al. (2013) found no differences between moderate and light intensity conditions on a modified Stroop task with a switching condition. Finally, Wang et al. (2013) found no differences in performance on the WCST between moderate, light and rest conditions.

At vigorous intensities, response times may be improved for certain aspects of cognitive flexibility, whereas accuracy may be impaired for others. For example, three studies assessed cognitive flexibility using the WSCT. Results and specific dependent outcomes variables varied between studies, but overall, performance was impaired during vigorous intensity exercise, compared to lower intensities or rest. When examined between-subjects, Dietrich and Sparling (2004) found an increase in the number of conceptual level responses and an increase in total number of errors when participants ran or cycled at an intensity ranging from moderate-to-vigorous (i.e., 70–80% HRmax), compared to rest. Similarly, Wang et al. (2013) found that participants made more perseverative errors, completed fewer categories and made fewer conceptual level responses under vigorous intensities compared to moderate, light and rest conditions. When examined within-subjects, performance also declined under vigorous exercise intensities compared to rest (Del Giorno et al., 2010). In contrast, the ability to switch between local and global processing may be enhanced. One study demonstrated overall improvements in response times, as well as quicker speed when switching between global to local trials, or vice versa, during vigorous intensities compared to rest (Pesce et al., 2007). Thus, similar to light intensity, vigorous intensity may impair certain aspects of cognitive flexibility and enhance others but effects are often dependent on the cognitive task used.

##### Integrative Summary of Intensity Effects on Executive Functions

Prior meta-analytic reviews have demonstrated that executive functions show beneficial and significantly larger effects than any other category of cognitive tasks (e.g., information processing, simple and choice reaction time, attention, memory) (Lambourne and Tomporowski, 2010; Chang et al., 2012). However, these effects were not dependent on exercise intensity, contradicting theoretical predictions where higher levels intensity are thought to hinder these higher-order cognitive processes (Yerkes and Dodson, 1908; Dietrich, 2003; McMorris et al., 2008; Dietrich and Audiffren, 2011). Notably, empirical acceptance that higher exercise intensities induce lower executive performance than moderate intensities has been mixed. However, conclusions drawn here and previously regarding executive function during exercise rely heavily on studies examining inhibitory control. Examining executive functions separately may lead to an ability to detect domain-specific intensity effects during exercise.

Here we find intensity-dependent effects for inhibition, such that moderate exercise intensity improves response time and moderate-to-vigorous intensity impairs accuracy. This pattern of results aligns with previous reviews, suggesting that complex cognitive tasks are more likely to be affected by exercise than simple tasks (McMorris and Graydon, 2000; Dietrich, 2006; Chang et al., 2012). This deterioration in inhibition under higher exercise intensities provides support for hypofrontality temporarily impairing executive function (Dietrich and Audiffren, 2011; Audiffren, 2016). Further, the declines in performance demonstrated during the initial minutes of exercise, or during heightened levels of physical load (as induced by intensity) may be the result of competing physiological resources. This competition may lead to reduced ability to inhibit prepotent motor responses and selectively attend and respond to target stimuli whilst ignoring goal-irrelevant stimuli. However, heterogeneity still exists and performance is often dependent on several moderating factors, including duration or fitness level.

In contrast, the impact of exercise intensity is less clear for working memory and cognitive flexibility. To assume these follow similar patterns demonstrated for inhibition with regards to effects of exercise intensity on response time and accuracy may be premature. For instance, here we find no clear systematic effects of exercise intensity on working memory performance, instead performance consistently declines across light, moderate and vigorous intensities. This is in contrast with inverted-*U* and hypofrontality hypotheses, but it aligns with previous work reporting a detrimental effect of moderate intensity on working memory tasks (McMorris et al., 2011). Different from working memory, cognitive flexibility shows clear intensity-dependent effects for accuracy and no consistent effects on response time. However, these effects appear to be moderated by the particular type of task used to assess performance (see Table 7). Furthermore, types of study designs used to measure working memory were more variable than cognitive flexibility and inhibition, which predominantly compared intensities of interest to a rest condition. Taken together, the findings are complex because the studies reviewed here suggest that executive functions are differentially sensitive to the effect of exercise intensity. Some cognitive processes are impaired at higher intensities (i.e., interference control, response inhibition), some remain fully efficient (i.e., response time), yet others show decrements at lower exercise intensities (working memory). Common across all executive functions is the ability to maintain and manage goals, and use those goals to bias ongoing processing (Friedman and Miyake, 2017). However, the extent to which exercise intensity effects one’s ability to use and apply goal representations when engaging inhibition, working memory or cognitive flexibility requires further research. In sum, future work on exercise intensity is needed before claims about specificity or generality of exercise effects on executive functions can be made.

#### The Impact of Exercise Intensity on Non-executive Functions

##### Attention

Effects of very light to light intensity exercise on attention are mixed (see Table 4). For example, among trained individuals, light-intensity exercise increased errors but did not influence response time on a sustained attention task relative to rest (Radel et al., 2018). Light-intensity exercise did not influence accuracy or response time on a visual oddball task relative to rest (Bullock et al., 2015). However, the remainder of studies measuring very light to light intensity exercise used lighter intensities as their control conditions, rather than using resting conditions as the control, limiting ability to draw conclusions.

Moderate intensity may improve response times, but either impairs or does not affect accuracy on attention tasks when compared to rest. For instance, moderate-intensity exercise speeded response times but increased errors on the SART (Radel et al., 2018). Similarly, moderate intensity speeded response times but reduced accuracy on visual and auditory oddball tasks (Yagi et al., 1999; Bullock et al., 2015). Moderate-intensity exercise increased false alarms on the CPT (Del Giorno et al., 2010) and speeded performance on the PSAT in one study (Quelhas Martins et al., 2013) but not another (Lambourne and Tomporowski, 2010). It speeded alerting, but not orienting or executive control of attention (Huertas et al., 2011). Finally, moderate-intensity exercise decreased response time with no effects on accuracy in two studies using a local global task requiring focusing of visual attention (Pesce et al., 2002), but did not influence speed of visual search (McMorris and Graydon, 1997). Taken together, moderate intensity exercise may improve speed of attentional processing with inconsistent effects on accuracy.

Vigorous to maximal intensities consistently improve attention. Of the six studies to include vigorous- to maximal-intensity exercise and rest conditions, four studies found improved response time (McMorris and Graydon, 1997; Pesce et al., 2004; Huertas et al., 2011; Quelhas Martins et al., 2013), with little evidence for impairments (Del Giorno et al., 2010), and one found no differences from rest (Ciria et al., 2019).

##### Motor Speed

Across intensities, exercise exerts inconsistent effects on motor speed, specifically reaction time (see Table 1). However certain trends are worth noting and exploring further. Exercise most often speeds reaction time for choice response time, during moderate-intensity exercise and most often slows reaction time for simple reaction time during vigorous-intensity exercise. Relative to rest, very light-intensity exercise has both speeded (Chmura et al., 1998) and not influenced (Davranche and Audiffren, 2004) response time, particularly choice response time. Very light-intensity exercise was also found to slow typing speed (John et al., 2009). Moderate-intensity exercise speeded choice response time across four studies relative to rest (Arcelin et al., 1998; Chmura et al., 1998; Davranche and Audiffren, 2004; Audiffren et al., 2008). Moderate-intensity exercise slowed simple response time in one study, in untrained individuals (Brisswalter et al., 1997). Vigorous-intensity exercise has speeded simple (Collardeau et al., 2001) and choice (Paas and Adam, 1991) response time relative to rest. Vigorous-intensity exercise has also slowed simple response time in four studies (Brisswalter et al., 1997; Collardeau et al., 2001; Ando et al., 2008, 2010).

##### Information Processing

The literature suggests that vigorous-intensity enhances information processing, whereas moderate-intensity may impair it (see Table 6). However, the research is limited to a few studies examining performance at each intensity level, compared to rest. Very light exercise enhanced visual threat detection (Shields et al., 2011). Moderate-intensity exercise improved critical flicker fusion thresholds in one study (Lambourne et al., 2010) but not another (Grego et al., 2004). Vigorous-intensity exercise improved information processing across three studies (Adam et al., 1997; McMorris and Graydon, 1997; Shields et al., 2011) and impaired performance in one study (Paas and Adam, 1991). The one study to evaluate information processing during near-maximal-intensity exercise found improved performance (McMorris and Graydon, 1997).

##### Memory

Across the range of exercise intensities, moderate intensity has most consistently been shown to improve memory, whereas lighter and heavier intensities have demonstrated mixed results (see Table 7). For example, very light and light-intensity exercise did not influence memory (Miles and Hardman, 1998; Hötting et al., 2016; Silvers et al., 2018), whereas moderate-intensity exercise enhanced memory (Grego et al., 2004; Labban and Etnier, 2011; Pyke et al., 2020; Wang et al., 2020). Vigorous intensity-exercise improved memory in five studies (Hötting et al., 2016; van Dongen et al., 2016; Keyan and Bryant, 2017a,b; Loprinzi et al., 2021), and not in five studies (van Dongen et al., 2016; Keyan and Bryant, 2017a; Silvers et al., 2018; Loprinzi et al., 2021). Vigorous-intensity exercise also increased memory interference (Crawford et al., 2021). Similarly, near-maximal-intensity exercise both improved and did not influence memory (Frith et al., 2017). The impacts of exercise intensity with regards to duration and time of memory encoding and retrieval are described in section “Findings by Exercise Duration”.

##### Integrative Summary of Intensity Effects on Non-executive Functions

Within tasks measuring non-executive perceptual-motor functions, such as motor speed and information processing, response time was the most common behavioral dependent variable. Of the studies that assessed speed (response time) as an outcome measure, results generally demonstrated faster information processing across all exercise intensities (very light to vigorous) and improvements in reaction time under moderate to vigorous exercise intensities. Consistent with previous conclusions, reaction time on simple tasks appears to be sensitive to acute exercise, but may not support the inverted-U hypothesis, where moderate intensities would yield greatest improvements. Indeed, previous work has suggested that moderate to vigorous intensity (40–79% VO_2_max or equivalent) may represent a threshold for improved speed of responding (McMorris and Hale, 2015). Moreover, there does not appear to be any significant effect of exercise intensity on accuracy of simple cognitive tasks, suggesting that non-executive cognitive processing is not particularly reliable or sensitive as a measure of cognitive performance during acute exercise (McMorris and Hale, 2012; McMorris et al., 2016).

Studies measuring attention during exercise were generally split between those finding improvements and those finding impairments or no effects at each level of exercise intensity. One consistent pattern noted was speeded response times during moderate to maximal exercise intensities, with inconsistent effects on accuracy. Such findings are consistent with studies focusing on motor speed, and suggest that moderate-intensity exercise speeds attentional processes.

Studies measuring memory processes during physical activity suggest that moderate-intensity exercise benefits memory, as does higher-intensity exercise, though less consistently. The memory literature is fairly circumscribed, especially given the variation in exercise intensity and duration and encoding and retrieval timing. Therefore, it is premature to say whether one intensity promotes memory over others, but overall, it seems that exercise is likely to improve memory, with little evidence of deleterious effects. The mechanism by which exercise putatively improves memory may involve increased catecholamine levels and ensuing synaptic plasticity within the hippocampus (Loprinzi, 2018), as well as hippocampal levels of brain-derived neurotrophic factor (BDNF). This interpretation is consistent with previous reviews on exercise and memory, which found that acute exercise exerted moderate to large effects on long-term memory (Roig et al., 2013; but see Loprinzi, 2018 for an example in which high intensity exercise does not impact long-term memory).

### Findings by Exercise Duration

#### The Impact of Exercise Duration on Executive Functions

##### Inhibition

When we investigate the impact of acute exercise on inhibition by duration, response times are enhanced, whereas accuracy is impaired during shorter duration exercise, between 0 and 15 min (see Table 1). For example, exercise up to 15 min enhanced response times on both motor response inhibition and interference control tasks across five studies (Davranche et al., 2009, 2015; Ando et al., 2013, 2014; Komiyama et al., 2017). In contrast, five studies demonstrated reduced inhibitory control, with three reporting increased error rates (Pontifex and Hillman, 2007; Ando et al., 2011: Komiyama et al., 2019) and two reporting both increased error rates and slowed response times (McMorris et al., 2009; Smith et al., 2016). Two studies demonstrated no effects (John et al., 2009; Labelle et al., 2013).

When we look at medium duration, exercise lasting 16–30 min exerts variable effects on response times and accuracy. Both aspects of inhibitory control have been shown to improve and decline in both motor response inhibition and interference control tasks. Specifically, five studies demonstrated improved response times (Davranche and McMorris, 2009; Joyce et al., 2009, 2014; Huertas et al., 2011; Olson et al., 2016; Finkenzeller et al., 2019), and one demonstrated combined improvements to both behavioral outcomes (Komiyama et al., 2016). In contrast, three studies demonstrated reduced accuracy, driven by increased error rates (Schmit et al., 2015; Olson et al., 2016; Finkenzeller et al., 2019), with one study demonstrating a more pronounced Simon effect, representing impaired response selection (Davranche and McMorris, 2009). Taken together, it appears acute exercise between 16 and 30 min may improve response time, or impair accuracy, but does not reliably slow response times. However, one study also demonstrated no effects (Huertas et al., 2011). As such, exercise intensity is likely a key factor moderating such changes to performance.

To date, long duration exercise, lasting 31 min or more, remains relatively unexplored limiting conclusions that can be drawn. More specifically, no studies have examined exercise lasting 31–45 min and two studies have examined performance at durations exceeding 45 min. Enhanced response times with increasing exercise duration was found during 60 min of vigorous intensity. Yet, reductions in accuracy were demonstrated under both very light and vigorous intensity conditions (Tempest et al., 2017). Conversely, 90 min of treadmill running at moderate intensity was shown to improve response times, with no change to accuracy, when compared to a light intensity condition (Giles et al., 2018). However, as there is limited work examining inhibition at extended durations, it is difficult to determine whether there is a threshold at which performance may begin to deteriorate.

##### Working Memory

Similar to intensity, there are no consistent effects of duration on working memory during exercise (see Table 2). Short-duration exercise, up to 15 min, has been shown to not influence working memory (Komiyama et al., 2017). Exercise lasting 16–30 min primarily resulted in no changes to working memory in four studies (Dutke et al., 2014; Komiyama et al., 2015, 2016, 2019), but improved response times during moderate intensity in one study (Quelhas Martins et al., 2013) and impaired accuracy during vigorous intensity in another (Komiyama et al., 2019). To date, no research has examined aspects of working memory during exercise lasting 31–45 min. Exercise lasting 46–60 min has demonstrated mixed results, with studies finding impaired accuracy (Tempest et al., 2017), enhanced speed (Rattray and Smee, 2013), or no changes in working memory performance (Travlos and Marisi, 1995).

##### Cognitive Flexibility

Within studies examining cognitive flexibility, exercise durations longer than 45 min have not yet been examined. At durations of 45 min or less, the relatively few number of studies and diversity of tasks used to measure varying aspects of cognitive flexibility makes it challenging to compare across studies (see Table 3). Short durations, up to 15 min, differentially influences aspects of cognitive flexibility. For instance, divergent thinking (Oppezzo and Schwartz, 2014) and ability to flexibly switch focus of attention from local to global (Pesce et al., 2003, 2007) may be improved during shorter bouts of exercise, whereas convergent thinking (Oppezzo and Schwartz, 2014) and task-switching may be impaired (Labelle et al., 2013). To note, these studies did not specifically report exercise duration and times were estimated from information reported in the methods. Exercise lasting 16–30 min resulted in impairments in accuracy on the WCST under vigorous intensity (Del Giorno et al., 2010; Wang et al., 2013), no differences under moderate intensity (Wang et al., 2013), and mixed results under light intensity (Del Giorno et al., 2010; Wang et al., 2013). Longer duration exercise, lasting 31–45 min impaired accuracy on the WCST at vigorous intensity (Dietrich and Sparling, 2004). More work is needed to specifically determine how factors such as intensity or duration may influence aspects of flexibility, such as perseveration and set-shifting, task-switching and switching focus on attention, as well as convergent and divergent thinking.

##### Integrative Summary of Duration Effects on Executive Functions

Similar to exercise intensity, exercise duration moderates executive function during exercise. Here we find negative effects for inhibition tasks performed between 0 and 15 min of exercise. These results were specific to accuracy, whereas response times improved up to 30 min, and declined past 60 min. These findings generally align with previous meta-analytic reviews demonstrating detrimental or negligible effects between min 11–20 min and beneficial effects after 20 min of exercise (Lambourne and Tomporowski, 2010; Chang et al., 2012). Given the few studies exploring cognitive flexibility and working memory across varying exercise durations, conclusions are still limited.

#### The Impact of Exercise Duration on Non-executive Functions

##### Attention

Exercise duration does not appear to consistently influence attention during exercise (see Table 4). Short-duration exercise, up to 15 min, enhanced aspects of attention in six studies (McMorris and Graydon, 1997; Yagi et al., 1999; Pesce et al., 2002, 2004; González-Fernández et al., 2017), impaired in one study (Wohlwend et al., 2017), and did not influence attention in two studies (McMorris and Graydon, 1997; Yagi et al., 1999), Exercise lasting 16–30 min improved attention in three studies (Huertas et al., 2011; Sanabria et al., 2011; Hüttermann and Memmert, 2014), impaired attention in three studies (Del Giorno et al., 2010; Hüttermann and Memmert, 2014; Cortney Bradford et al., 2019), and did not influence attention in two studies (Hüttermann and Memmert, 2014; Ciria et al., 2019). Exercise lasting 31–45 min enhanced speed of attention when moderate in intensity (Bullock et al., 2015; Radel et al., 2017, 2018; Sanchis et al., 2020), but increased errors when light and moderate in intensity (Radel et al., 2018). In contrast, one study demonstrated no influence on attention (Lambourne et al., 2010).

##### Motor Speed

During moderate-intensity exercise, and exercise durations between 15 and 90 min, we see that exercise most often speeds reaction time for choice response time. Exercise most often slows reaction time for simple reaction time, for vigorous-intensity exercise, and very short (less than 15 min) and long (more than 90 min) durations (see Table 5). The majority of studies used durations less than 15 min. Of these, one found speeded choice response time (Arcelin et al., 1998), three found slowed simple response times (Brisswalter et al., 1997; Ando et al., 2008, 2010), and one found no difference (Arcelin and Brisswalter, 1999). Of the three studies of 15–30 min exercise duration, two found speeded choice response times (Paas and Adam, 1991; Davranche and Audiffren, 2004), and one found no difference (Davranche and Audiffren, 2004).

##### Information Processing

Performance on perceptual tasks have only been assessed across a few exercise durations, limiting conclusions (see Table 6). Exercise between 20 and 40 min exerts variable effects on information processing, enhancing performance in three studies (Adam et al., 1997; Lambourne et al., 2010; Shields et al., 2011), but impairing in one (Paas and Adam, 1991). Exercise of longer durations, particularly in the second and third hour of exercise of 180 min durations, impaired information processing (Grego et al., 2004; see Table 2). However, to date, durations less than 20 min and between 40 and 180 min remain unexplored.

##### Memory

Memory appears to be improved or unaffected across the spectrum of exercise durations (see Table 7). In addition to intensity and duration, the timing of encoding and retrieval is essential to consider. Encoding occurred from 2 days before exercise to during exercise, and retrieval occurred during exercise to 1 week after exercise.

Ten studies evaluated the influence of exercise on encoding before exercise and retrieval during or after exercise. For encoding 2 days before exercise, 20–25 min of vigorous-intensity exercise enhanced memory for central details 2 days following exercise when memory was reactivated during exercise (Keyan and Bryant, 2017a). Exercise did not influence memory when not reactivated, nor for peripheral details or intrusive memories. Thirty five minutes of vigorous-intensity exercise improved cued recall 2 days after exercise for information encoded 4 h before exercise, but not immediately before exercise (van Dongen et al., 2016). For encoding 10 min before exercise, 30 min of vigorous-intensity exercise enhanced memory 24 h after exercise, but not 20 min after exercise (Hötting et al., 2016). The same study found no effects of very light to light-intensity exercise on memory.

For encoding immediately before exercise, 10 min of very light- to vigorous-intensity exercise did not influence cued recall 2 days after exercise, but increased intrusive memories (Keyan and Bryant, 2017b). Thirty minutes of moderate- to vigorous-intensity enhanced verbal memory 35 and 60 min as well as 24 h after encoding (Labban and Etnier, 2011; Wang et al., 2020) and old/new recognition 80–90 min after encoding (Pyke et al., 2020). Fifteen minutes of near-maximal-intensity, short-duration exercise enhanced 20-min and 24-h delayed verbal memory as well as prospective memory when performed before encoding, but not during or after encoding (Frith et al., 2017). Fifteen minutes of vigorous-intensity exercise also increased memory interference 5 min after exercise, relative to rest (Crawford et al., 2021). Twenty minutes vigorous-intensity exercise did not influence incidental or intentional encoding immediately or 30 min after exercise (Loprinzi et al., 2021), but enhanced verbal recall through encoding and retrieval in Experiment 1 and consolidation in Experiment 2 (Loprinzi et al., 2021).

In addition to Frith et al. (2017) described above, three studies evaluated the influence of encoding during exercise and retrieval during or after exercise. Moderate-intensity exercise improved map recognition between the first and second of 3 h exercise (Grego et al., 2004). Verbal memory was better when encoded and retrieved during 11 min exercise and when encoding and retrieved during rest than when encoding during exercise and retrieved during rest and vice-versa, providing evidence for state-dependent learning (Miles and Hardman, 1998). Finally, 20 min very light-, light-, and vigorous-intensity exercise did not influence immediate or 1-week delayed memory (Silvers et al., 2018).

##### Integrative Summary of Duration Effects on Non-executive Functions

The majority of studies focusing motor speed show speeded responses on SRT, particularly after 15 min of exercise. Likewise, exercise improves information processes after 20 min of exercise, but performance declined after 2 and 3 h of exercise. On the other hand, exercise has been generally shown to improve attentional processes up to 15 min of exercise, after which the effects become more variable. Overall, it appears attentional processes improve very early in the exercise bout, whereas perceptual-motor processes improve after some time. However, more work spanning short to long durations is need to determine specific time points at which perceptual-motor and attentional processes are impacted.

For memory, exercise duration as well as the timing of encoding and retrieval are essential to consider in terms of sequence of events. A recent meta-analysis encompassing the present articles, as well as those in which exercise occurred prior to encoding, suggests that although exercise during memory encoding did not influence retrieval, short-duration exercise tended to impair memory relative to the control (Loprinzi, 2019). Exercise consolidation enhanced episodic memory, particularly for long-duration exercise during early consolidation, and short-duration exercise during late consolidation. Around the time of that meta-analysis, a handful of studies have begun to better disentangle the effects of encoding and retrieval timing by administering encoding before, during, and after exercise, and retrieval a relatively short and long while after exercise (Frith et al., 2017; Pyke et al., 2020; Loprinzi et al., 2021). These studies have broadly suggested that the mechanism by which exercise enhances memory may work through encoding, consolidation, and retrieval. Future research should take a similar approach, but also systematically vary duration and intensity. At present, the literature suggests that exercising between learning and retrieving information improves memory at best, and does not influence memory at worse. Given exercise’s benefits to stress and mood (Basso and Suzuki, 2017), it is likely to benefit learning contexts.

## What Is Next?

As we have seen in the preceding sections, the impact of exercise on executive and non-executive processes varies dramatically depending on the specific cognitive domain, as well as by intensity and duration. In the following section, we discuss several moderating factors that may contribute to these mixed findings, highlight existing gaps in our knowledge and propose future directions for work in this field.

### Factors That Impact Cognition During Exercise

#### Participant Characteristics

##### Fitness Level

Certain participant characteristics may affect cognition during exercise. One important characteristic to consider is participant’s fitness level. To date, studies have compared young adults of varying fitness levels (see Supplementary Table 1 for participant characteristics for all studies). For example, studies examining the impacts of fitness on executive functions found that exercise did not influence inhibition among higher- or lower-fit individuals, but increased error rate more so in lower- than higher-fit individuals (Labelle et al., 2013). Interestingly, majority of studies examining motor and perceptual processes during exercise have used physically fit individuals. For example, within studies assessing motor speed, nearly all extant studies included physically fit individuals, whose VO_2_max averages fell above the 50th percentile, often above the 75th percentile (Kaminsky et al., 2015). The one study to compare simple response time during exercise between trained and untrained individuals found that exercise at all intensities ranging from light to vigorous slowed simple response time in untrained individuals, but this effect dissipated at moderate and vigorous-intensity exercise in trained individuals (Brisswalter et al., 1997). Indeed, populations such as endurance athletes may differ from the general population. However, to date still few studies have compared cognitive function during exercise between lower and higher fit individuals, or between sedentary individuals and/or athletes of varying fitness levels. Thus, future work should consider how this participant characteristic may guide or impact research questions.

##### Psychological Factors

Future work should also consider the psychological factors that are at play in realistic exercise scenarios (e.g., anticipatory anxiety before athletic performance, cognitive stress during military operations) that may influence motivation in lab-based research. For example, acute anxiety experienced during exercise has been shown to mitigate declines in inhibitory control under long duration, high intensity exercise (Cantelon et al., 2019). Additionally, research has demonstrated that mental resource allocation, perception of effort and prefrontal cortex activation are differentially affected when exercise end-point is known vs. unknown (Radel et al., 2017; Wingfield et al., 2019), yet it remains unknown how such anticipation may influence cognitive function during exercise. Given that a motivating factor for much of the research in this field is to characterize performance decrements that could lead to costly performance outcomes (e.g., game-losing play, or life or death decisions), basic work should seek to emulate the emotional and motivational factors that may influence performance in applied settings.

#### Methodological Factors

##### Dependent Outcome Measures: Speed vs. Accuracy

Another factor that contributes to the heterogeneous patterns of results observed within the acute exercise-cognition literature is the dependent outcome variable measured. Speed (response time) and accuracy of cognitive performance tend to show differential patterns of results. For example, improvements to inhibition are largely driven by faster responses, whereas decrements are driven by changes in response accuracy. Previous meta-analyses have demonstrated similar effects, such that when accuracy was the dependent variable, results were significantly different to those when response time was the dependent variable (McMorris et al., 2011; McMorris and Hale, 2012). Inconsistent findings with regards to accuracy within the exercise-cognition literature may be driven by use of cognitive tasks primarily designed to measure speed of processing (i.e., flanker, simple, go/no-go, simple, and choice reaction time) (McMorris, 2016), or the result of an inability to detect exercise-induced changes due to ceiling effects often demonstrated in the healthy young populations sampled (McMorris and Hale, 2012). However, given that response time is more consistently influenced than accuracy across various cognitive domains, interpretations are limited. It is possible that different mechanisms may be contributing to the changes to response time or accuracy during exercise (McMorris and Hale, 2015). Thus, if changes to response times or accuracy are elicited at distinct or differing physiological thresholds, ability to adequately assess changes to both speed and accuracy in a single study may be limited. Future work should continue to explore these differential effects on response time and accuracy, as ability to detect even slight deteriorations of such processes could allow us to better predict potential negative outcomes (life or death, losing play in a game).

##### Exercise Modality

Exercise modality has also been shown to differentially impact cognition during exercise. For example, within the executive domain, studies demonstrating impairments in working memory used treadmill, while those demonstrating improvements or no differences employed cycle ergometry. Previous meta-analytic reviews have revealed that exercise modality is an important factor in determining cognitive function during exercise, where running was shown to be related to declines in performance and cycling with improvements (Lambourne and Tomporowski, 2010). Inconsistent results based on exercise modality may be attributable to differences in the physical effort required during running vs. cycling. For instance, walking and running require balance and control of body posture. Thus, negative effects of simultaneous exercise on, say, working memory, may be due to the attentional conflict between coordination of bodily movement and executive control. Performing cognitive tasks during exercise inherently creates a dual-task environment, but these dual-task effects may be more pronounced depending on the exercise modality (see Supplementary Table 1 for exercise modality used in all studies in this review). However, given that the vast majority of studies utilize cycling compared to running, walking, or other forms of aerobic exercise, more research is needed to further establish whether specific relationships exist between exercise modality, intensity and cognitive domains.

### Additional Gaps and Future Directions

#### Unified Methods for Prescribing Intensity

Exercise intensity is often considered the most important component of exercise prescription as it represents the magnitude of metabolic stress on the body. Poor quantification or inadequate control of intensity likely contributes to the heterogeneity of results in exercise-cognition relationships. Furthermore, prescription and categorization of exercise intensity often varies from study to study. For example, 60% HRmax may be classified by authors as moderate, whereas using the ACSM guidelines followed here, this intensity is considered to be within the light range. Hence, the field needs unified methods so results can be more easily compared across studies. It has been argued that intensity should be determined in relation to the aerobic threshold, which might allow us to better understand the physiological and biochemical factors that contribute to changes during exercise. This may also confer better understanding of exercise intensity effects on cognition for exercise prescription. Relatedly, cognitive testing at rest, either before exercise or during a separate resting day, is essential to interpret results across studies, and as such, future studies should aim to test cognition at rest compared to during exercise.

#### Intensity Gaps

**FIGURE 1:**
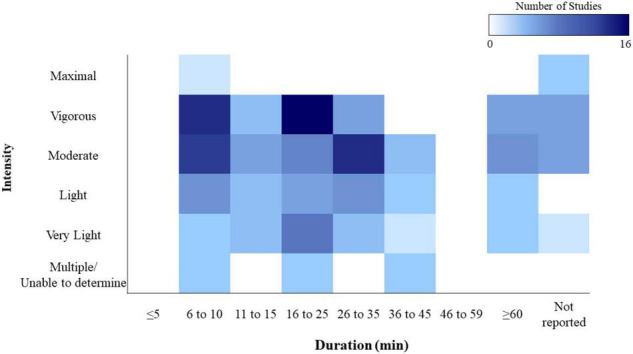
Heat map illustrating the number of studies assessing changes across all cognitive domains by each intensity and duration. Studies using exercise intensities spanning multiple intensity categories (i.e., moderate-vigorous, moderate-varied) are included as “multiple/unable to determine.” Exercise duration excludes warm-up time. 73 total studies represented. Table inspired by Pontifex et al. (2019).

As evidenced in *section (“What Has Been Done?”)*, research spanning the matrix of exercise intensities and durations is incomplete (see Figure 1). Notably, across all cognitive domains, very few studies have explored performance under maximal effort conditions. This extreme exercise intensity poses methodological challenges, however, understanding how cognition is impacted under such conditions may have applied relevance for certain populations (i.e., military personnel or athletes). Such examination may provide insights into when and/or how fatigue leads to declines in cognitive performance.

In line with this notion, future work should continue to examine how cognitive performance may change at, or near, physiological transition points. For example, it appears that inhibition accuracy may begin to decline above the ventilatory threshold (VT). Indeed, reduced accuracy has been demonstrated in studies prescribing target intensities at vigorous, or near the upper boundary of the moderate intensity range [i.e., 60% VO_2_peak; 60% MAP (∼70–82% HRmax)] (Garber et al., 2011; Olson et al., 2016; Finkenzeller et al., 2019) and in previous work using an incremental exercise protocol (Da Silva et al., 2017). In these studies, reduced accuracy was driven by inability to avoid strong prepotent responses, or distraction from task-irrelevant information. Thus, one thought is that increasing exercise intensity may reduce the ability to effectively maintain and use goal representations to bias competition from conflicting information. Higher levels of physical stress leads to shifts in attentional deployment, with increased internal (associative) focus of attention at the expense of attentional resources available for external (dissociative) focus (Tenenbaum, 2007). Research has demonstrated that at intensities above VT individuals often report increases in associative thoughts relating to physical sensations, or interoceptive cues, that accompany the metabolic changes due to exercise (i.e., breathing rhythm, muscle fatigue, heart rate, and temperature) (Ekkekakis et al., 2011; Da Silva et al., 2017). These attentional shifts mirror the theorized redistribution of resources from PFC to motor areas, as well as observed activation of regions associated with autonomic regulation (i.e., insular cortex) demonstrated at increasing exercise intensities (Dietrich and Audiffren, 2011; Audiffren, 2016; Fontes et al., 2019). Thus, future work should examine whether specific physiological thresholds must be exceeded before declines in cognitive function are revealed, and specifically where accuracy of executive functions may begin to deteriorate.

#### Duration Gaps

Future work should also look to fill the gaps in our understanding of exercise duration, particularly for endurance exercise. Changes in cognition over relatively short durations are pertinent to the general population aiming to meet the Physical Activity Guidelines (American College of Sports Medicine, 2018), or athletes who perform in discrete playing periods. However, changes in cognition during longer duration exercise are essential to understand for endurance athletes as well as military personnel and emergency responders, who must remain cognitively intact in prolonged, physically demanding situations. While many studies look at cognition as a function of exercise intensity, very few do so for exercise duration. To date, this area of the field remains largely unexplored, as evidenced in Figure 1, where the majority of studies focus on durations of 45 min or less.

Furthermore, the lack of systematic and consistent findings with regards to longer exercise durations may be due to timing of data sampling. Time-averaged cognitive performance does not fully capture potential temporal dynamics of cognitive functioning throughout the exercise bout. For example, during a prolonged bout performance may increase or decrease at varying time points. As such, new theoretical perspectives have been proposed to explain how top-down (cognitive and physical efforts) and bottom-up processes (bodily sensations) may act in parallel of arousing mechanisms to dynamically influence cognitive performance across time (see Schmit and Brisswalter, 2018 for review of this fatigue-based neurocognitive perspective). However, given the scarce number of studies investigating performance at durations extending beyond 45 min, theoretical predictions and current understanding of time-dependent changes remain understudied.

Finally specific reporting of duration parameters will improve our ability to draw conclusions about specific effects due to exercise duration. For instance, within the domain of attention, majority of experiments performed did not report total exercise duration. Lack of reporting duration parameters, including total time spent exercising and minutes spent in warm-up and at prescribed intensities, as well as specific timing of when cognitive tasks were administered is problematic and makes it difficult or impossible to interpret results on how duration impacts cognition. Similar to developing unified methods for determining exercise intensity, common methodological factors should be reported across future work.

### Statistical Quantification of Exercise Effects

Finally, the narrative nature of this review allowed for a more nuanced exploration of how cognition is impacted during exercise that has potentially been overlooked in prior research. For instance, Chang et al. (2012) concluded that exercise intensity does not influence cognitive performance, but here we see that intensity-dependent effects may depend on the cognitive task type, exercise duration and/or fitness level. These complex effects are difficult to capture using meta-analytic techniques. However, lack of systematic and quantitative comparison also limits the conclusions that can be drawn. Here, we aimed to highlight potential areas where future work may be useful in order to enhance conclusions that can be drawn from meta-analysis. Thus, as empirical work examining cognitive changes during exercise grows, new meta-analytic reviews will be essential in identifying reliable exercise-induced effects.

### Limitations

The present review only included studies prescribing aerobic exercise. Yet, the majority of empirical studies measuring cognition during exercise often employ bouts of aerobic activity. This may be due to the fact that previous work in this field has largely been driven by understanding and enhancing performance of athletes, as well as law enforcement and military personnel, who often operate under such aerobically demanding conditions. However, given that alternate forms of exercise (i.e., HIIT, resistance, coordinative, etc.) are gaining popularity, both within the general public and research community, it is important for future work to characterize exercise-cognition interactions beyond aerobic exercise. Specifically, understanding cognitive changes elicited at physiological thresholds may be well-suited for incremental or HIIT exercise protocols, where strictly controlled exercise intensities allow for more precise measurement of intensity-induced changes.

In depth examination of underlying neural mechanisms facilitating cognitive changes during acute bouts of exercise was not within the scope of this review. Additionally, not all of the studies reviewed here conducted objective measurement of the mechanisms involved in the exercise effects reported. However, characterizing how underlying neural changes influence cognition, such as changes in cerebral blood flow or electrical potential, is important and will be necessary in allowing the field to further develop and refine current theories. As future research continues to explore exercise-cognition interactions, it will be important to tie neurological and physiological mechanisms to changes in cognitive function.

## Conclusion

The current review summarized the critical characteristics of literature examining cognitive changes during acute bouts of aerobic exercise. First, we characterized what aspects of cognition have been explored during exercise and common cognitive tasks used. Across cognitive domains, we find more evidence for exercise impacting speed over accuracy of responding. Future work should consider how choice of cognitive tasks and populations sampled may impact ability to detect changes in behavioral outcomes of interest. In line with this notion, we suggest that adopting standardized methods of prescribing and reporting exercise parameters would be advantageous for the field.

Next, to date, extant literature has largely focused on examining one sub-component of executive function during exercise, namely inhibition. Working memory and cognitive flexibility are two other important components of executive function. Conclusions about how inhibition is impacted during exercise might not generalize to working memory and cognitive flexibility. Regardless of executive or non-executive domain, under shorter durations and light to moderate intensities cognitive performance may not be drastically impacted. Higher intensity and longer duration exercise may impair certain aspects of cognition, but literature in this area remains sparse. Finally, information presented here may provide translational application for sports performance or individuals working under states of physical exertion, such as endurance athletes or first responders and military personnel.

Overall, the effects on cognition during exercise are likely mixed due to methodological differences referenced above, but also because exercise exerts its effects through multiple mechanisms. Research should continue to characterize cognitive changes during exercise, as well as the mechanisms that drive such changes, in order to refine and develop the theories of exercise-induced changes to cognition. This will help us to develop tools to predict cognitive changes during physical exertion.

## Author Contributions

JC and GG were responsible for conceptualizing and designing the review and interpreting relevant literature. JC was responsible for write-up of the manuscript. Both authors contributed to manuscript revision, as well as read and approved the final manuscript.

## Conflict of Interest

The authors declare that the research was conducted in the absence of any commercial or financial relationships that could be construed as a potential conflict of interest.

## Publisher’s Note

All claims expressed in this article are solely those of the authors and do not necessarily represent those of their affiliated organizations, or those of the publisher, the editors and the reviewers. Any product that may be evaluated in this article, or claim that may be made by its manufacturer, is not guaranteed or endorsed by the publisher.

## Supplementary Material

Supplementary Table 1 Complete summary of studies included in this review.
